# Conversion of Polyethylene to Low-Molecular-Weight Oil Products at Moderate Temperatures Using Nickel/Zeolite Nanocatalysts

**DOI:** 10.3390/ma17081863

**Published:** 2024-04-18

**Authors:** Hyungjin Cho, Ahyeon Jin, Sun Ju Kim, Youngmin Kwon, Eunseo Lee, Jaeman J. Shin, Byung Hyo Kim

**Affiliations:** 1 Department of Materials Science and Engineering, Soongsil University, Seoul 06978, Republic of Korea; hyungjincho@soongsil.ac.kr (H.C.); jshin@ssu.ac.kr (J.J.S.); 2 Department of Green Chemistry and Materials Engineering, Soongsil University, Seoul 06978, Republic of Korea

**Keywords:** nickel nanocatalyst, upcycling, hydrogen, polyethylene

## Abstract

Polyethylene (PE) is the most widely used plastic, known for its high mechanical strength and affordability, rendering it responsible for ~70% of packaging waste and contributing to microplastic pollution. The cleavage of the carbon chain can induce the conversion of PE wastes into low-molecular-weight hydrocarbons, such as petroleum oils, waxes, and natural gases, but the thermal degradation of PE is challenging and requires high temperatures exceeding 400 °C due to its lack of specific chemical groups. Herein, we prepare metal/zeolite nanocatalysts by incorporating small-sized nickel nanoparticles into zeolite to lower the degradation temperature of PE. With the use of nanocatalysts, the degradation temperature can be lowered to 350 °C under hydrogen conditions, compared to the 400 °C required for non-catalytic pyrolysis. The metal components of the catalysts facilitate hydrogen adsorption, while the zeolite components stabilize the intermediate radicals or carbocations formed during the degradation process. The successful pyrolysis of PE at low temperatures yields valuable low-molecular-weight oil products, offering a promising pathway for the upcycling of PE into higher value-added products.

## 1. Introduction

Polyethylene (PE) is the polymer produced in the largest quantity, with an annual production of 100 million tons. PE is used in packaging materials such as plastic bags, beverage caps, and straws due to its low cost and high chemical stability [1,2,3,4,5,6]. The usage of PE as a disposable item results in a massive amount of PE waste, which accounts for approximately 34% of total plastic waste [7], leading to significant environmental and social issues [8]. Most PE waste is either landfilled or incinerated, resulting in problems such as landfill capacity limitations, resource loss, and additional environmental pollution [6,9,10,11]. Mechanical recycling through melt reprocessing allows for the reutilization of about 16% of plastics but suffers from property degradation [9,12,13,14]. To overcome these drawbacks, chemical recycling methods, known as upcycling, have gained much attention [10,14,15,16,17].

Upcycling primarily involves the thermal degradation of PE, cracking the hydrocarbon chains into low-molecular-weight products such as oil, naphtha, and gas, and thus creating higher-value materials [8,18,19,20]. Since PE is composed of C-C bonds without specific functional groups, the thermal degradation temperature is relatively high at around 400 °C, necessitating a considerable energy input [21]. To mitigate the high energy consumption in the degradation of PE, recent studies have aimed to lower the reaction temperature [8] by using hydrogenation catalysts [6,22,23,24,25].

The addition of hydrogen during the thermal degradation of PE facilitates C-C bond cleavage, enhancing upcycling efficiency [12,25,26,27]. The hydrogenation catalysts with a high efficiency can be selected by a volcano plot. The Sabatier principle posits that the ideal catalyst ought to establish interactions of moderate affinity with both reactant species and resultant products. The catalyst’s binding strength should be sufficiently robust to engage with reactants effectively, yet suitably feeble to facilitate the dissociation and subsequent formation of products. The volcano plot, rooted in this principle, delineates the correlation between a catalyst’s catalytic activity and its binding affinity in a systematic manner [28]. In the volcano plot, noble metals such as Pt, Rh, and Ru can be the most effective catalysts for hydrogenation reactions [1,27,29,30,31]. However, considering the high cost and limited reserves of these catalytic elements, the development of cost-effective metal-based nanocatalysts is in high demand [27]. Nickel catalysts exhibit a good catalytic activity among non-noble metals, with an abundant availability and low cost, making them suitable for industrial-scale reactions [31]. Several groups have investigated nickel-based thermal degradation catalysts for PE upcycling using supported nickel precursors via impregnation or deposition methods. Dionisios and coworkers utilized Ni/SiO_2_ catalysts for PE degradation, achieving diesel yields of 40–70%, which is comparable to the diesel yield achieved when using Pt- and Ru-based catalysts [27]. Rui Cai and coworkers obtained gas-phase hydrocarbon yields of up to 89.1% through C-C bond cleavage when using Ni- and Ru-based catalysts [32]. Pioneering studies have shown the applicability of Ni-based catalysts for PE upcycling but still require a high reaction temperature or produce a low oil yield.

**Figure 1:**
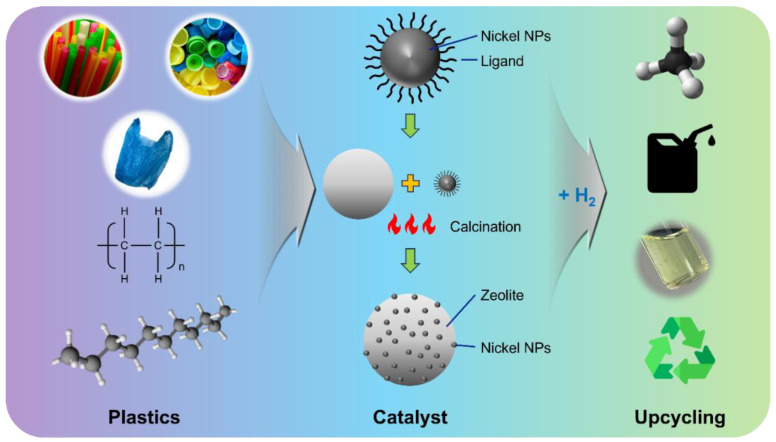
Schematic diagram of upcycling of PE using nickel/zeolite nanocatalysts.

Herein, we synthesized uniform-sized nickel nanoparticles and incorporated the particles into zeolite supports for PE-upcycling catalysts (Figure 1). The nanometer-sized grain size of the Ni nanoparticles of the catalysts offers advantages in terms of a high surface area to increase the catalytic efficiency [33]. Large-sized nanoparticles among a non-uniform nanoparticle ensemble have a low surface atom ratio, and thus nanoparticle-based catalysts with a broad size distribution usually show weak catalytic properties. Nickel nanoparticles are supported on porous zeolites [30,34,35], which serve not only as a support but also as a Lewis acid site to enhance C-C bond cleavage by initiating and stabilizing radicals [1,29,36,37,38]. We conducted PE thermal degradation at a relatively low temperature of 350 °C by adjusting the hydrogen pressure and catalyst quantity.

## 2. Materials and Methods

### 2.1. Materials

Nickel(II) acetylacetonate (Ni(acac)_2_, 90%), Dioctyl ether (99%), trioctylphosphine (90%), oleylamine (70%) and low-density polyethylene were purchased from Sigma-Aldrich (St. Louis, MO, USA). Zeolite HY (zeolite Y modified with hydrogen) was purchased from Thermo Scientific (Waltham, MA, USA).

### 2.2. Methods

#### 2.2.1. Synthesis of 10 nm Nickel Nanoparticles

The monodisperse Ni nanoparticles were synthesized by following a previous report [39]. The synthetic procedure for the preparation of the nickel–oleylamine complex was commenced with a reaction between 1.55 g of oleylamine and 0.257 g of Ni(acac)_2_ at 100 °C for a duration of 30 min under an inert atmosphere. Subsequently, 5 mL of dioctyl ether was injected into the complex solution, which underwent degassing for 1 h at 100 °C under vacuum conditions. Following this step, 0.3 mL of trioctylphosphine was carefully added to the solution under an argon atmosphere. The temperature was gradually elevated to 250 °C at a rate of 2 °C/min and maintained at this level for 30 min. The color of the solution transitioned from bluish-green to black at approximately 200 °C, indicative of the formation of nickel nanoparticles. Upon completion of the reaction, the product was swiftly cooled to room temperature. Nanoparticles were then precipitated by adding a mixture of toluene and excess ethanol in a 1:3 ratio. Finally, the obtained nanoparticles were dispersed in hydrophobic solvents such as hexane or toluene for further analysis and applications.

#### 2.2.2. Preparation of Polyethylene Degradation Catalyst

Synthesized nanoparticles were impregnated onto a zeolite support. Specifically, nickel nanoparticles were synthesized and dispersed onto Y-zeolite, with a weight ratio of 1:10, where 0.5 g of nickel nanoparticles was impregnated onto 5 g of zeolite, which had an approximate size of 1 μm. The nanoparticles, bearing phosphine ligands, were dispersed in hexane, a non-polar solvent. The zeolite support was then immersed in the dispersion, followed by sonication for 30 min and stirring at 1000 rpm. The sonication and vigorous stirring were repeated several times to ensure uniform impregnation of the nanocatalyst onto the zeolite surface. Subsequently, the solvent was removed using a rotary evaporator, and the ligands attached to the particles were eliminated by calcination in air atmosphere at 450 °C for 3 h using a muffle furnace.

#### 2.2.3. Polyethylene Upcycling

PE upcycling was conducted using a batch reactor with mechanical stirring, wherein the pressure and catalyst amount were controlled. To ensure the reaction took place under inert condition, the reactor was purged with $N_{2}$ gas for 10 min to remove oxygen from the interior. Subsequently, $H_{2}$ gas was introduced and evacuated up to 15 bar, repeating this process five times to transition the interior to a hydrogen atmosphere. Pressure and temperature were measured using pressure gauges and temperature sensors built into the batch reactor. The reaction was carried out at 350 °C for 2 h under $H_{2}$ atmosphere. Pressure-related experiments were conducted by varying the H_2_ pressure to 1 bar, 10 bar, and 20 bar. The catalytic efficiency was measured by varying the catalyst amount to 1%, 5%, and 10% by weight while fixing the $H_{2}$ pressure at 10 bar and the amount of PE at 5 g.

#### 2.2.4. Characterization of Materials

Transmission electron microscopy (TEM) and energy dispersive spectrometer (EDS) analyses were conducted using a JEOL JEM-2100F instrument (Tokyo, Japan) operating at an accelerating voltage of 200 kV. Fourier transform infrared spectroscopy (ATR FT-IR) measurements were performed using a VERTEX 70 FT-IR spectrometer (Bruker, Ettlingen, Germany). Gel Permeation Chromatography (GPC) was carried out using a Waters GPC system with tetrahydrofuran (THF) as the solvent and a flow rate of 1.0 mL/min. Gas chromatography mass spectroscopy (GC-MS) analysis was performed using the Chromatec Crystal 9000 instrument from CHROMATEC (Yoshkar-Ola, Russia) with a VF-5MS column. Thermogravimetry analysis (TGA) was performed using a Metler Toledo H8-1430KR (Columbus, OH, USA), under N_2_ atmosphere with a heating rate of 10 °C/min. Brunauer–Emmett–Teller analysis (BET) was performed using a Quantachrome Instruments ASIQM00002200-7X (Boynton Beach, FL, USA). X-ray diffraction (XRD) was performed using a Bruker D2 phase.

## 3. Results and Discussion

### 3.1. Analysis of Nanoparticles and Catalysts

The Ni nanoparticles were synthesized by thermal decomposition of Ni(acac)_2_–oleylamine complexes in dioctyl ether solvent at 250 °C. Dioctyl ether used as a solvent is degassed before reaction to remove moisture. The formation of Ni(acac)_2_–oleylamine complexes enables the thermal decomposition of Ni precursors at a moderate temperature [39]. The color change from light green (for the Ni(acac)_2_) to bluish-green (for the Ni(acac)_2_–oleylamine) may be attributed to the coordination number changes of Ni from 4 to 6 [40].

**Figure 2:**
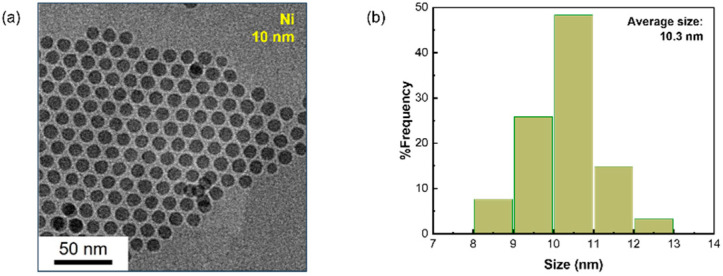
(**a**) TEM image of 10 nm nickel nanoparticles and (**b**) corresponding size distribution.

The synthesized uniform nickel nanoparticles have a size of 10 nm (10.3 nm ± 0.8 nm) based on TEM analysis using ImageJ program (Figure 2a). TEM images of Ni nanoparticles synthesized using trioctylphosphine as a surfactant are depicted in Figure 2a. The TEM image and corresponding size distribution histogram reveal the high monodispersity of the particles (Figure 2b).

We mixed the nanoparticles with zeolite before conducting a 450 °C calcination process. Since the surface of the nanoparticles is surrounded by long-chain hydrocarbon ligands, which make it difficult for reactants and catalyst materials to gain access, we conducted a calcination process to remove the ligands.

**Figure 3:**
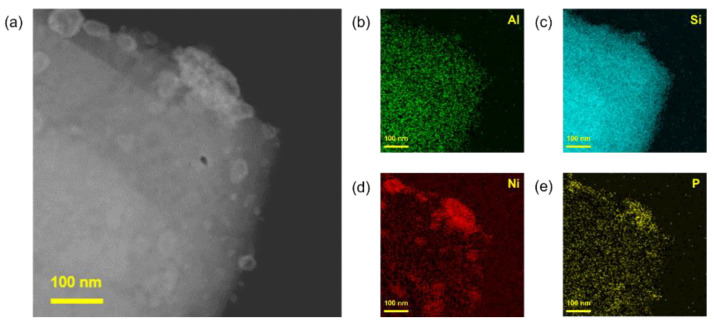
STEM image of Ni on Y-zeolite; weight ratio 1:10. (**a**) Ni on Y-zeolite. EDS data for each element: (**b**) Al, (**c**) Si, (**d**) Ni and (**e**) P.

Scanning transmission electron microscopy (STEM) imaging and energy dispersive analysis (EDS) of the calcined catalysts showed that the Ni nanoparticles were successfully impregnated onto the zeolite support (Figure 3). The presence of nickel nanoparticles on the zeolite surface was confirmed through EDS analysis. It is presumed that the relatively high amount of phosphorus of the EDS data occurs because the phosphorus of trioctylphosphine used as ligands for the nanoparticles remains after calcination. The size of the nickel nanoparticles on the zeolite surface ranged from approximately 10 to 30 nm, indicating that some slight agglomeration occurred during the calcination process, but they still exhibited a smaller size distribution compared to conventional catalysts [41,42].

The X-ray diffraction (XRD) pattern exhibits a Y-zeolite crystal structure with small and broad peaks at 43°, presumably due to the fact that Ni nanoparticles have a polycrystalline structure with a grain size of ~3 nm (Figure S2) [39]. The small and broad XRD peaks of Ni nanoparticles resulting from the polycrystalline nature are enshrouded by the strong XRD peaks from Y-zeolite with a grain size of about 1 μm.

Through Brunauer–Emmett–Teller (BET) analysis, we measured the surface area of the Y-zeolite before and after nanoparticle loading. The surface area of the pure Y-zeolite was 1283 m^2^/g, and decreased to 976 m^2^/g after loading 10 nm Ni nanoparticles onto the zeolites, blocking the pores with the incorporated nanoparticles (Figure S1). Both pure Y-zeolite and Ni/zeolite catalysts show a similar average pore size diameter, 3.83 nm, indicating that the structure of the zeolite remained intact even after calcination at 450 °C.

The dispersion (D) of catalysts is calculated by the following equation [43,44,45],

$$D=\frac{6V}{A\times d}\times 100\left(\%\right)$$

where V is the atomic volume of a Ni atom, A is the surface density of a Ni atom on the Ni(111) plane, and d is the average particle size. The dispersion of our Ni-based nanocatalysts is 8.53%.

### 3.2. Analysis of PE Upcycling

#### 3.2.1. Effect of Hydrogen Pressure on PE Upcycling

**Figure 4:**
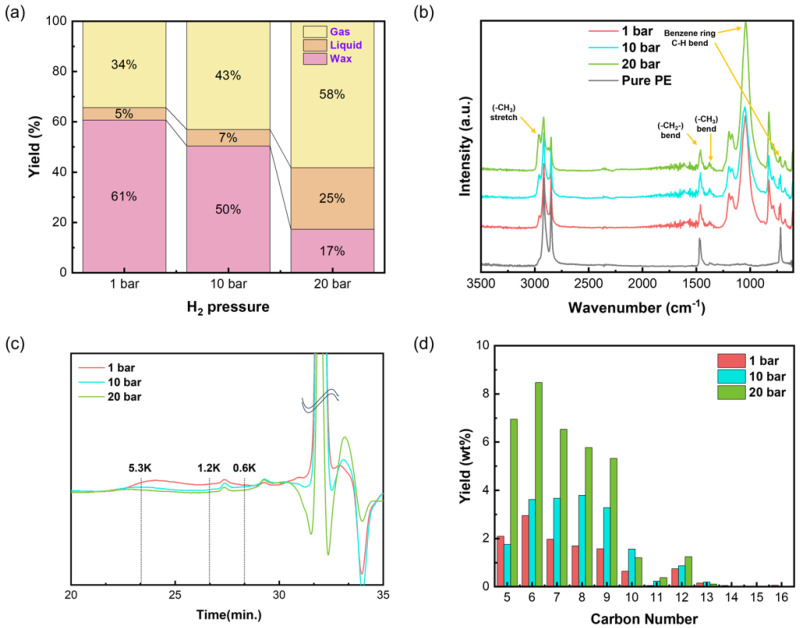
Effect of hydrogen pressure on the PE upcycling. (**a**) Percentage yield of each phase, (**b**) FT-IR spectra, (**c**) GPC data, and (**d**) GC-MS carbon number data of PE upcycling product under different hydrogen pressures of 1 bar, 10 bar, and 20 bar. The PE upcycling was conducted at 350 °C for 2 h, and the catalyst loading amount, when compared to the polymer, was 10 wt%.

To investigate the impact of hydrogen pressure on the PE pyrolysis when using nickel on Y-zeolite catalysts, we carried out the process with different hydrogen pressures of 1 bar, 10 bar, and 20 bar. The obtained products consisted of gas, liquid, and wax phases. Under the 1 bar hydrogen pressure, PE catalytic pyrolysis resulted in 34% wax-phase, 5% liquid-phase, and 61% gas-phase products. Upon increasing the hydrogen pressure to 10 bar, the percentage of gas-phase product increased to 43%, while that of wax-phase product decreased to 50%. We achieved a 25% yield of liquid-phase oil product under the condition of a hydrogen pressure of 20 bar (Figure 4a). The results suggest that increasing pressure enhances the C-C bond cleavage reaction of PE. As hydrogen pressure increases, the molecular weight of PE decreases, leading to a higher yield of liquid-phase and gas-phase hydrocarbons.

FT-IR analysis has the capability of differentiating between PE polymers and pyrolysis products with a low molecular weight. The C-H stretch peaks in the range of 2900–3000 cm^−1^ for hydrocarbons exhibit slightly different peak positions depending on the surrounding environment. The C-H stretch peak for -CH_2_- in the middle of the hydrocarbon chain appears at 2920 cm^−1^, while the peak for -CH_3_ at the chain end is located at 2950 cm^−1^ [46,47]. The difference originates from the weakening of the C-H bond strength when increasing the number of electron-donating alkyl groups. By comparing the peaks at 2950 cm^−1^ and 2920 cm^−1^, it is possible to determine the qualitative ratio of carbons at end sites of the hydrocarbon chain. In Figure 4b, the FT-IR spectra of PE polymers with a very low number of chain ends show marginal peaks at 2950 cm^−1^ corresponding to -CH_3_. The intensity of the -CH_3_ stretch peaks becomes more prominent for the PE upcycling product, indicating the degradation of long polymer chains and an increase in chain ends. Similarly, the -CH_3_ bend peaks at 1376 cm^−1^ are more distinct in the products after hydrogenation compared to PE, and the intensity of the peak increases at a higher hydrogen pressure. The FT-IR analysis indicates that higher hydrogen concentrations lead to the production of shorter hydrocarbons with lower molecular weights. FT-IR spectra also show the presence of aromatic hydrocarbons.

We also conducted gel permeation chromatography (GPC) of the obtained liquid and solid products to identify the molecular weight range of the pyrolyzed product under different hydrogen pressures. When the catalytic pyrolysis was conducted under 1 bar hydrogen pressure, the resulting product showed a GPC peak starting at 22 min, which is expected to contain molecules with a molecular weight ranging from 1000 Da to 5000 Da. In contrast, when the degradation was carried out under 10 bar and 20 bar pressure, almost no peak could be identified at the same range, suggesting that degradation occurred into even lower molecular weight hydrocarbons at higher pressures. The system GPC peak at around 32 min for the pyrolysis product at the reaction condition of 20 bar hydrogen pressure indicates the generation of a significant amount of low-molecular-weight hydrocarbons (Figure 4c).

The obtained liquid products were analyzed using gas chromatography-mass spectrometry (GC-MS). The results of the analysis of the products obtained at 1 bar, 10 bar, and 20 bar pressures are shown in Figure 4d. The liquid products contained hydrocarbons ranging from C5 to C16, with a predominance of products closer to C9. Considering that the hydrocarbons from C5 to C12 are utilized as gasoline, the liquid products obtained at 1 bar, 10 bar, and 20 bar pressures primarily consist of gasoline-range hydrocarbons with percentages of 97.75%, 98.97%, and 99.69%, respectively [9,48]. The quantitative analysis indicates that our catalyst exhibits a high selectivity for hydrocarbons in the gasoline range. Additionally, the negligible amount of hydrocarbons with carbon numbers higher than C13 suggests that the catalyst effectively breaks down long-chain PE into hydrocarbons within the fuel range.

**Scheme 1:**
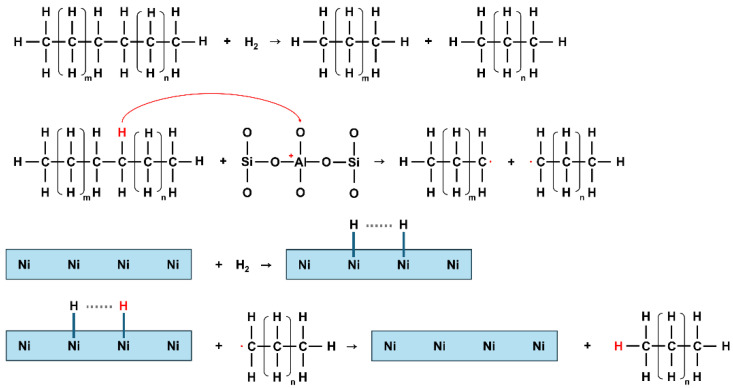
Suggested PE degradation mechanism using zeolite and nickel nanoparticles. Symbols highlighted in red indicate atoms or electrons involved in reactions.

The FT-IR, GPC, and GC-MS analyses indicate that a higher degradation rate is observed under the condition of a high hydrogen pressure, suggesting that the hydrogen-involved process may play a key role in the pyrolysis mechanism. The PE degradation mechanism occurs in accordance with Scheme 1. The degradation process of PE using the nickel/zeolite catalysts involves the formation of radicals on the PE polymer chain through Lewis-acidic Y-zeolite [49], the adsorption of hydrogen onto the nickel surfaces, and the generation of short chains by reacting adsorbed hydrogen and radicals. When inferring that more degradation occurs at a higher hydrogen pressure, one sees that the adsorption of hydrogen or the reaction between adsorbed hydrogen and radicals may be the rate-determining step. The hydrogen binding energy of nickel and platinum is 3.07 eV and 0.094 eV, respectively. The higher binding energy of nickel compared to platinum suggests that the chemisorption process could contribute to differences in reaction rates [50,51]. The effect of hydrogen pressure varies with different catalyst systems, potentially due to differences in adsorption and desorption energy. Further studies are required to gain a deeper understanding of the catalytic mechanism [52].

#### 3.2.2. Effect of Catalyst Amount on PE Upcycling

To investigate how the quantity of the catalysts affects the degradation of PE, PE pyrolysis experiments were conducted by changing the Ni/Y-zeolite catalyst to PE ratio, as follows: 0 wt%, 1 wt%, 5 wt%, and 10 wt%. The reactions were carried out at 350 °C and 10 bar hydrogen pressure for 2 h.

**Figure 5:**
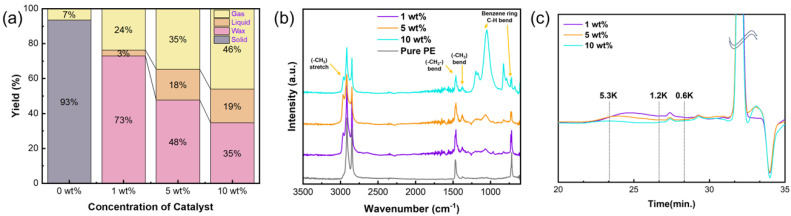
(**a**) Percentage yield of each phase, (**b**) FT-IR spectra, and (**c**) GPC data of PE upcycling product at different catalyst wt%: 1 wt%, 5 wt%, and 10 wt%.

The pyrolysis experiment involving a control of the catalyst amount elucidated the effect of catalysts on the pyrolysis temperature. Without catalyst, the pyrolysis temperature of 350 °C proves inadequate for breaking down the resilient C-C bond, as shown in Figure 5a. After the reaction at 350 °C for 2 h, 93.4% of PE polymers remain in solid form, with only 6.6% of polymer chains undergoing cleavage and converting to gas form. Conversely, in the presence of catalysts under the same reaction conditions, most polymer molecules are converted to gas, liquid, and wax forms. The high degradation temperature of PE is confirmed by TGA (Figure S3). TGA data of PE polymers under N_2_ atmosphere indicate that PE degradation initiates at 400 °C, which is 50 °C higher than the degradation temperature observed in the presence of the Ni/Y-zeolite catalysts. These findings underscore the significant role of the Ni-based catalysts in reducing the activation energy required for C-C bond cleavage.

The portion of oily liquid-phase product among the products obtained after pyrolysis of PE with 1 wt% of catalysts was only 2% (Figure 5a). This indicates that with low amounts of catalysts, the C-C bond cleavage reaction does not occur sufficiently to produce oil. With a higher catalyst loading of 10 wt%, the oil-phase liquid fraction escalated to 7% of the products resulting from the pyrolysis reaction, alongside a noticeable increase in gas-phase products, presumably due to the catalyst-induced end-cleavage reactions. Considering that the oil-phase product is the most valuable out of the three phases, the nickel/Y-zeolite catalyst impregnated with 10 wt% seems to offer economic benefits. Adjusting catalyst quantities proves conducive to specific product outcomes: a large wt% of the catalyst favors the natural gas production of PE, while a low wt% of the catalyst ratio would be preferable for obtaining wax-phase products.

The extent of the hydrocarbon chain cleavage of PE was assessed via the IR spectrum of the product. An increase in the proportion of carbon at end sites compared to pure PE was confirmed through a comparison of peaks at 2950 cm^−1^ and 2930 cm^−1^ (Figure 5b). Interestingly, a distinctive peak positioned at 1035 cm^−1^ was observed for the IR spectrum of catalytically pyrolyzed product with a high wt% of the nickel/zeolite catalysts. The catalytic pyrolysis of PE also yielded aromatic compounds, evident from the appearance of the benzene ring, as indicated by the 1035 cm^−1^ peak in the IR spectra.

The degradation of PE to low-molecular-weight carbon is also confirmed by the solubility change and GPC analysis. While the reactants, high-molecular-weight PE, are insoluble in any solvent, such as tetrahydrofuran (THF), the products, hydrocarbon molecules resulting after the catalytic pyrolysis reaction, are well dissolved in the THF solution, indicating the cleavage of PE polymer chains. The GPC data of upcycled PE using 1 wt% nickel/zeolite catalysts show a strong peak in the 1000 to 5000 Da range. The highest molecular weight of pyrolyzed products is 5300 Da. Based on the GPC analysis, we conclude that a significant portion of the PE polymer molecules has been cleaved into low-molecular-weight hydrocarbons. Furthermore, the wax form of the solid product implies a significant reduction in mechanical strength compared to the initial PE. The phenomenon is corroborated by the GPC results, which show the low molecular weights of the product.

## 4. Conclusions

In this study, nickel-based catalysts for PE upcycling were prepared by loading nickel nanoparticles onto zeolite supports. PE polymer chains were successfully decomposed into gas, liquid, and wax phases at 350 °C by using the nickel/zeolite catalysts. As the hydrogen pressure and catalyst amount increased, the proportion of gas- and liquid-phase products increased. PE upcycling using the nickel/zeolite nanocatalysts enabled the conversion of plastic waste into high-value liquid products at a relatively moderate pyrolysis temperature. Overall, our research highlights the potential of nickel-nanoparticle-based catalysts supported on zeolites to efficiently upcycle PE at lower temperatures, thereby contributing to the advancement of sustainable strategies for plastic waste management.

## Data Availability

Data are contained within the article and Supplementary Materials.

## Supplementary Materials

The following supporting information can be downloaded at: https://www.mdpi.com/article/10.3390/ma17081863/s1, Figure S1. Absorption (black)-desorption (red) nitrogen isotherms of the (a) Pure Y-zeolite (surface area 1307.960 m^2^/g), (b) Nickel on Y-zeolite (surface area 1079.580 m^2^/g). Figure S2. X-ray diffraction spectra of nickel on Y-zeolite (red) and Y-zeolite (black). Due to nickel’s polycrystalline structure, its XRD peak appears smaller and less prominent compared to that of zeolite, which forms a crystalline structure. Figure S3. Thermogravimetric curve of pure LDPE at a heating rate of 10 °C/min under N_2_ atmosphere.
