# Supplementary material for: Conversion of Polyethylene to Low-Molecular-Weight Oil Products at Moderate Temperatures Using Nickel/Zeolite Nanocatalysts
Source: Materials (Basel). 2024 Apr 18;17(8):1863. doi: 10.3390/ma17081863 (PMC11051393; doi:10.3390/ma17081863)
Supplement: Supplementary file 1 [file materials-17-01863-s001.zip › materials-2919104-supplementary.pdf]

Supplementary materials for:

Conversion of Polyethylene to Low-Molecular-Weight Oil Products at Moderate Temperatures Using Nickel/Zeolite Nanocatalysts

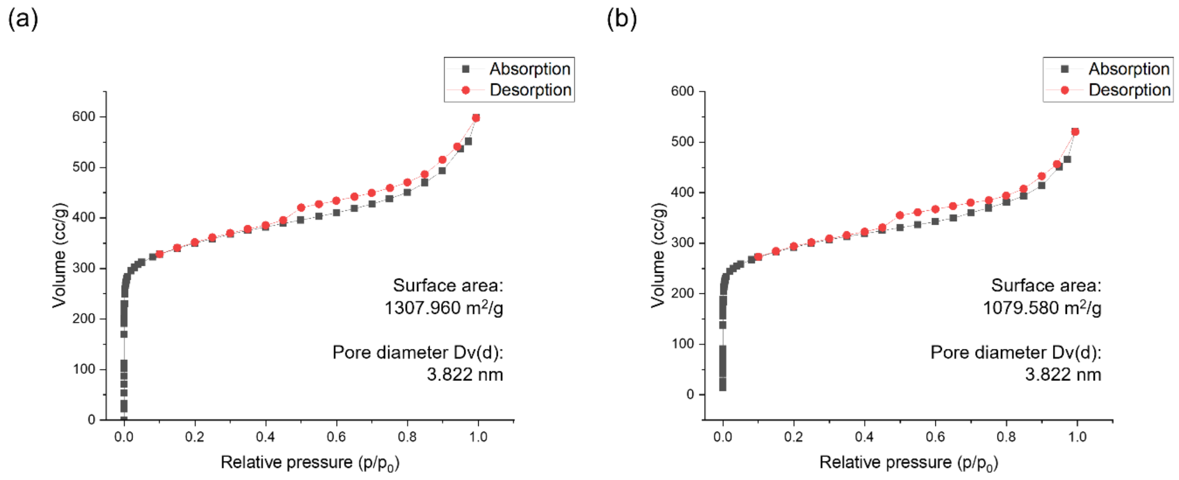

Figure S1. Absorption(black)-desorption(red) nitrogen isotherms of the (a) Pure Y-zeolite (surface area 1307.960 m^2/g), (b) Nickel on Y-zeolite (surface area 1079.580 m^2/g).

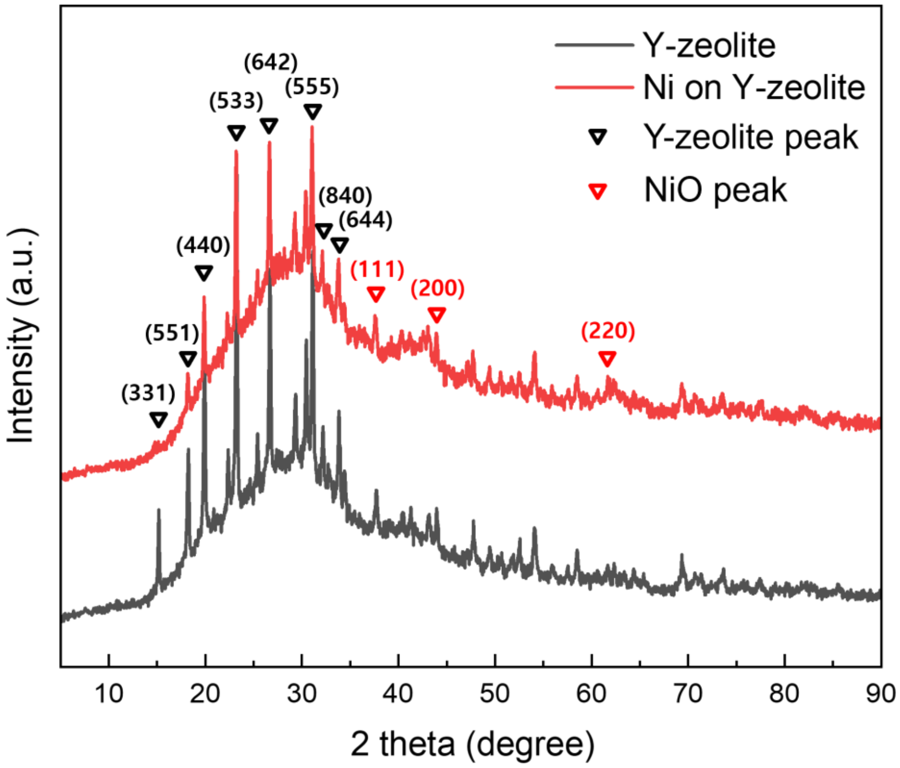

Figure S2. X-ray diffraction spectra of nickel on Y-zeolite(red) and Y-zeolite(black). Due to nickel's polycrystalline structure, its XRD peak appears smaller and less prominent compared to that of zeolite, which forms a crystalline structure.

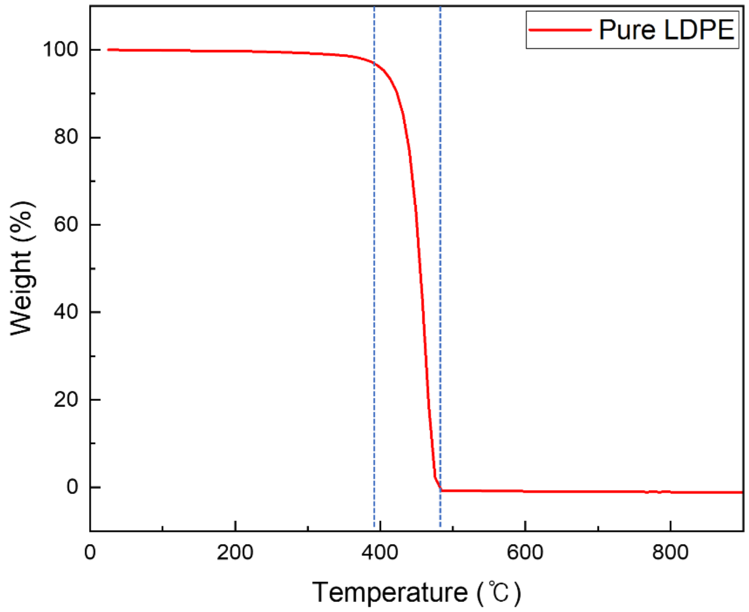

Figure S3. Thermogravimetric curve of pure LDPE at a heating rate of 10 ^{o}C/min under N_2 atmosphere.
